# Theoretical Accounts of Gulf War Syndrome: From Environmental Toxins to Psychoneuroimmunology and Neurodegeneration

**DOI:** 10.1155/2002/418758

**Published:** 2002-01-01

**Authors:** Eamonn Ferguson, Helen J. Cassaday

**Affiliations:** School of Psychology University of Nottingham Nottingham NG7 2RD UK

**Keywords:** cytokines, interleukin-1, stress, Gulf War Syndrome, Pavlovian conditioning, neurodegeneration

## Abstract

Non-specific illness includes a wide variety of symptoms: behavioural (e.g., reduced food and water intake), cognitive (e.g., memory and concentration problems) and physiological (e.g., fever). This paper reviews evidence suggesting that such symptoms can be explained more parsimoniously as a single symptom cluster than as a set of separate illnesses such as Gulf War Syndrome (GWS) and chronic fatigue syndrome (CFS). This superordinate syndrome could have its biological basis in the activity of pro-inflammatory cytokines (in particular interleukin-1: IL-1), that give rise to what has become known as the ‘sickness response’. It is further argued that the persistence of non-specific illness in chronic conditions like GWS may be (in part) attributable to a bio-associative mechanism (Ferguson and Cassaday, 1999). In the case of GWS, physiological challenges could have produced a non-specific sickness response that became associated with smells (e.g., petrol), coincidentally experienced in the Persian Gulf. On returning to the home environment, these same smells would act as associative triggers for the maintenance of (conditioned) sickness responses. Such associative mechanisms could be mediated through the hypothalamus and limbic system via vagal nerve innervation and would provide an explanation for the persistence of a set of symptoms (e.g., fever) that should normally be short lived and self-limiting. We also present evidence that the pattern of symptoms produced by the pro-inflammatory cytokines reflects a shift in immune system functioning towards a (T-helper-1) Th1 profile. This position contrasts with other immunological accounts of GWS that suggest that the immune system demonstrates a shift to a Th2 (allergy) profile. Evidence pertaining to these two contrasting positions is reviewed.

## 1. Introduction

There is now a range of evidence to suggest that as well as physiological challenges and trauma both psychological parameters and environmental factors have an important role to play in the presentation and severity of disease [9]. These effects are not limited to self-reports of symptoms and illness but also include changes in physiological parameters [60,101]. Such interactions are plausible because sensory information can influence physiological parameters (e.g., immune cell trafficking or reactivity) via neurological mediation. This can be either central via limbic system structures or more direct via brainstem structures (see [69, 70]). Research has tended to focus on these mechanisms more in relation to established conditions, such as cancer [3], viral recurrence [57], the common cold [15] and physiological mechanisms such as wound healing [59], rather than in conditions where the diagnostic criteria are less well established (cf. [8,105]). The spectrum of symptoms reported in the latter conditions is often referred to as functional or medically unexplained [8,18]. In this review we examine, and attempt to integrate, the different theoretical perspec-

ISSN 0953-4180/01/02/$8.00 © 2001/2002 – IOS Press. All rights reserved tives taken on one such condition: Gulf War Syndrome (GWS). Recent theory and empirical evidence suggests that medically unexplained symptoms (MUSs) may be accounted for by a single symptom dimension. If so, in addition to an improved understanding of GWS in and of itself, the theoretical analysis presented should give insights into other conditions (e.g., chronic fatigue syndrome, irritable bowel syndrome). Given the diversity of theoretical perspectives taken on GWS we shall summarize the strengths and weakness of each and then present a theoretical synthesis in terms of a psychoneuroimmunological account.

The spectrum of symptoms observed in GWS is broad and diverse involving cognitive (e.g., memory), behavioural (e.g., anorexia), emotional (e.g., anxiety) and physiological symptoms (e.g., fever). Thus a complete theoretical account of this diversity will inevitably involve interactions between neurological, immunological and behavioural processes. The main body of this paper is structured around theoretical accounts of GWS based on separate neurological, psychological, behavioural, immunological and psychiatric mechanisms. These separate theoretical accounts are evaluated against two main criteria: (1) their ability to account for the symptom diversity and (2) their ability to account for the persistence of symptoms. Then an integrated perspective based on psychoneuroimmunology is presented.

While there is some debate as to whether the term syndrome is applicable to the array of symptoms reported by Persian Gulf War veterans, the term Gulf War Syndrome (GWS) is used in this paper for consistency with most published studies. Before exploring the different theoretical accounts, this review will first address the nature of the illness experienced on return from the Persian Gulf.

## 2. GWS – does it exist?

Like other multi-system syndromes (e.g., chronic fatigue syndrome, CFS), GWS is a controversial disorder. In part, the controversy surrounding these disorders arises because the breadth of reported symptoms makes both differential diagnosis and the identification of aetiologic agents problematic (see [16]). With respect to GWS it has been argued that the pattern of symptom clusters seen in veterans is qualitatively similar to the pattern reported by healthy era controls (e.g. [21,52, 61]) and that no clear diagnosis can be made from the clinical reports (see [53]). In this case, there would be nothing unique about GWS (but see e.g. [96]).

This is based on the idea that illness is best viewed categorically; however, it is equally valid to view illness dimensionally (cf. [120]). In the case of GWS, this would mean that the same underlying biological processes could result in symptoms in healthy controls (with a similar pattern but expressed at a lower level). Consistent with this, all published papers on GWS have demonstrated that veteran groups report higher levels of symptoms than controls (including era veterans) (e.g. [51,85,109,113]). There is also evidence to suggest that patients with different psychiatric diagnoses produce similar patterns of symptom clusters in response to diagnostic symptom assessments [111, 112]. However, it does not necessarily follow that clusters of symptoms that are similar share the same underlying causal mechanism [28]. For example, colds and flu show similar patterns of symptoms, but their causal agents are different, although, in this case, aspects of the biological mechanisms (immune system activation) are similar.

The second argument to suggest that GWS does not exist is based on the similarity of post-war death rates, hospitalization, birth defects and suicide rates in veterans and controls [36,67]. However, it has been argued that these results may reflect a number of methodological and statistical biases [39]. One such bias is the ‘healthy warrior effect’, or the notion that deployed troops are going to be healthier (physically and mentally) than non-deployed troops. If this were the case, then it would be expected (other things being equal) that the non-deployed troops would be more likely to present with illness than the deployed ‘healthier’ group (discounting the effects of their war zone experiences). If this potential bias is acknowledged, it follows that no difference between the comparison groups (deployed versus non-deployed) would mean that the deployed ‘healthier’ groups were nevertheless more ill (but see [17,35,37,40,56]).

Finally it has been argued that the spectrum of symptoms documented in GWS is similar to that seen in other wars (e.g., [49,105]). However, Haley [38] argues that: (1) the conditions present in other wars (e.g., the American Civil War) were fundamentally different to those in the Persian Gulf; (2) the diagnostic tests for the physical causes of illness were not as developed as they are today; and (3) for certain wars (i.e., the Vietnam War) assessments took place a long time after the cessation of hostilities. Thus it has been suggested that the post-war syndromes seen after other conflicts are not comparable [38].

In sum, Gulf War veterans report increased levels of symptoms that have persisted for 10 years. While there is not sufficient evidence to state that there is a unique 'syndrome' (cf. [118]), the extent of distress suffered by these veterans is just cause to try and explain their condition. With an improved understanding of the mechanisms associated with GWS, we will be in a position to begin to understand the similarities and differences between GWS and other functional syndromes such as chronic fatigue syndrome (CFS).

### *2.1. GWS and other functional somatic syndromes*

GWS shares many symptomatic features with other functional somatic conditions (e.g., chronic fatigue syndrome [CFS] or irritable bowel syndrome [IBS]) and as such any theoretical account that can offer a reasonable explanation for GWS should also be considered as a potential candidate explanation for these conditions (cf. [8]). For example, both factor analytic [18] and theoretical work [8,120] examining the co-morbidity of a variety of functional syndromes such as CFS and IBS has suggested that these can be explained by a single higher order factor (see also [29]). This is consistent with the possibility of a shared bio-psychosocial mechanism [8]. Figure 1 provides a schematic representation of the link between the factors identified by Haley et al. [43] for GWS and Deary's [18] analysis of other functional syndromes.

In addition to specifying the underlying dimensions in different functional syndromes, there is also a need to understand temporal variability. For many chronic conditions patients will report that they have good days and bad days. Is there similar temporal variability in GWS? Most studies examining the breadth of symptoms associated with GWS have tended to make single time interval assessments, so, at present, virtually nothing is known about the temporal patterning of symptoms in GWS. One study measured the symptoms of GWS at two time points separated by 2–4 years [86], finding no significant changes in the level of reported symptoms. By contrast, other research, exploring 'anniversary reactions' in veterans, has demonstrated systematic variability in subjective health states: following their return from the Gulf, veterans report more severe symptoms during the same months that they experienced their most traumatic experience in the Gulf [79]. This is consistent with the position that symptoms associated with GWS show temporal variation associated with wartime triggers/events. However, the temporal resolution of this work is broad and day-to-day variability has yet to be explored [29].

### *2.2. Criteria for an adequate account of GWS and other (MUSs)*

The following criteria are proposed for evaluating theoretical explanations of GWS. First and foremost, a complete account should be able to account for the breadth, persistence and variability of the symptoms. If an account of GWS can explain the breadth, persistence and variability in symptoms then the following additional criteria should also be applied to judging the plausibility of that account: (1) the proposed account should suggest testable hypotheses, (2) it should relate experiences in the Gulf to the veterans' current symptoms, and (3) a full explanation should include a biological mediating pathway for the symptoms experienced.

### *2.3. Theoretical explanations of GWS*

Five accounts of GWS are described below and grouped in terms of their proposed mechanisms of operation: (1) psychological, (2) neurological, (3) immunological, (4) behavioural and (5) psychoneuroimmunological. Of course they are not mutually exclusive. For example, stress (psychological) is used to explain how conditions in the Gulf may have assisted in allowing organophosphates (OPs) to enter the central nervous system (CNS) (but see [103]). However, in this case OP poisoning is the immediate cause of the neurological damage seen in GWS, not stress per se [38,41].

## 3. Psychological mechanisms

A range of psychological accounts of GWS (e.g., stress, post-traumatic stress disorder and psychiatric co-morbidity) are reviewed below.

### *3.1. Stress*

There is already good evidence that the experience of stress is related to a variety of physical health parameters. Psychological stress activates the hypothalamic-pituitary-adrenal (HPA) and sympathetic-adreno-medullary (SAM) axes. It is argued that the subsequent release of corticosteroids influences disease states (see [26]). Again in such cases, stress is not necessarily causal as such, but (though the evidence on this is mixed) stress may act to moderate biological systems (e.g., the blood-brain-barrier)

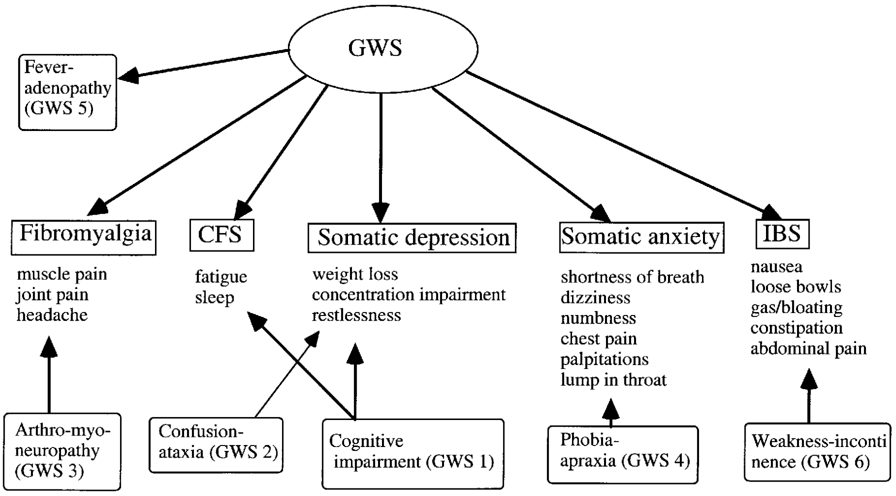

Note: GWS1, 2, 3, 4, 5 & 6 refer to Haley et al.'s (1997 [43]) syndromes. Fibromyalgia, CFS, Somatic depression, Somatic anxiety and IBS from Deary (1999 [18]).

Fig. 1. GWS: single or multi-faceted syndrome?

to make them more susceptible to disease provoking agents [45]. However, effects on the permeability of the blood-brain-barrier have not been consistently reproducible [103].

There are a number of papers that have explored the role of the self-reported levels of battlefield stress in relation to levels of currently reported symptoms of GWS. Some suggest that self-reports of battlefield stress have limited explanatory power (see [109]), providing, for example, little account of the diversity of symptoms [86]. However, others have demonstrated that, compared to healthy controls, veterans with CFS and psychiatric co-morbidity report more stressful events 6 months after the war [31]. These authors further find that veterans with CFS or with CFS and multiple chemical sensitivity (MCS) do in fact report more combat stress exposures (though in this case there was no control for psychiatric co-morbidity). It has also been demonstrated that reports of exposure to particular battlefield stressors (e.g., the belief that biological or chemical weapons were used, cf. [30]) were associated with reports of current symptom levels [80].

The evidence reviewed above suggests that battlefield stress cannot provide a complete account of current illness. However, there are a number of issues that need to be considered before its role is dismissed altogether. First, stress experienced during environmental exposures in the Gulf could have made biological systems more vulnerable to disease provoking agents [45] (but see [103]) and any such interactive effects would not be detectable (only) through the retrospective recall of (moderating) events. Second, through hormonal modulation, chronic stress can affect the function of the HPA axis as well as neurological structures. For example, in a rat model it is possible to examine the effects of blocking corticosteroid action in the hippocampus, compared with the more widespread effects produced by intracerebroventricular injection of selective antagonists. There is data to suggest that these endogenous steroid hormones are homeostatically regulated (with circadian fluctuation) by both hippocampal and extrahippocampal corticosteroid receptors [14]. Additionally it has been found that fluctuations in corticosterones can produce neurotransmitter disturbances, for example in hippocampal 5-HT receptor function, consistent with the effects of chronic stress in producing depression [76].

Stress and stress related hormones have also been demonstrated to affect cognitive functions dependent on the hippocampus. For example, experimentally, both chronic psychosocial stress (in subordinate tree shrews) and long-term cortisol treatment affect memory processes that rely on normal hippocampal functioning [81]. The hole board paradigm was used to provide a tests of spatial reference memory (known to be sensitive to the effects of hippocampal lesions, see e.g. [107]). These impairments were furthermore associated with a tendency towards reduced hippocampal volume, as measured by MRI, consistent with the finding that stress and glucocorticoids can cause structural changes in hippocampus [74].

In human subjects, stress has similarly been found to affect cognitive processes. For example, a naturalistic study using examination stress in students found that changes in (salivary) cortisol during exam times were correlated with increases in the perceived level of stress and some impairment in attention and (short-term) memory [115]. Thus stress can have interactive neuropsychological effects (see also section on *Neurological mechanisms* below). However, on its own, stress is not sufficient account of the breadth, persistence and temporal variability of symptoms seen in GWS.

Thus while the role of stress may be limited it can operate synergistically with other vulnerability factors to produce symptoms.

### *3.2. Post traumatic stress disorder* (*PTSD*)

The effects of the long-term chronic stress experienced by veterans could be especially serious in those with a diagnosis of PTSD. There are essentially two arguments pertaining to PTSD and GWS. The first concerns the differential prevalence rates of PTSD in Gulf War veterans compared to controls. The second concerns whether or not the levels of symptoms reported by Gulf War veterans can be accounted for by variation in levels of PTSD.

With regard to the first issue a number of studies have reported a higher prevalence of PTSD in Gulf War veterans than in controls (e.g. [7,72]). However, it has been argued that this higher prevalence of PTSD represents a false positive error rate due to the relative sensitivities and specificities of the assessments used for the diagnosis of PTSD [38].

A number of studies have shown that PTSD is related to the reporting of current physical symptoms of GWS (see [7,25,122]). The strength of this relationship is not large enough to support the view that PTSD provides a complete account of GWS. However, even given Haley's [38] critique of the assessment of PTSD in veterans, the consistency in the results in relation to symptom reporting suggests that some PTSD-related mechanism contributes to GWS (see [50]).

### *3.3. Psychiatric co-morbidity*

Can the physical symptoms reported in GWS be explained by psychiatric co-morbidity in veterans? A number of studies have examined these issues using a variety of diagnostic tools (see [62,108]). A number of authors writing recently agree that psychiatric diagnosis and co-morbidity, while apparent and important (e.g., increased levels of depression and PTSD), do not reflect a major cause of the physical symptoms reported in GWS [62,121].

### *3.4. Summary of psychological mechanisms*

Three psychological mechanisms have been discussed (stress, PTSD and psychiatric co-morbidity). It is concluded that battlefield stress may have acted to make veterans more susceptible to OP poisoning (but see [103]). Chronic stress (maybe manifest in PTSD) might even have led to neuropsychological changes. Thus stress needs to be considered in models of GWS, but as a moderating factor rather than as sufficient cause in itself. Neither levels of stress, PTSD, nor psychiatric co-morbidity, can account for the breath of symptoms seen in GWS, or for their persistence. However, PTSD may account for some degree of temporal variability (e.g., anniversary reactions).

## 4. Neurological mechanisms

A number of hypotheses focus on neurological damage. These suggest that environmental toxins produce alterations in the CNS that can result in permanent illness. Three of the main accounts will be explored here: (1) organophosphate induced delayed-polyneuropathy (OPIDP), (2) depleted uranium poisoning (DU) and (3) MCS.

### *4.1. OPIDP*

The symptoms of GWS include various signs consistent with impaired CNS functioning, from sleep disturbance, affective and cognitive problems, to headaches and migraines, even blackouts and dizziness in some [16]. It has been argued that these symptoms can be explained by exposure to organophosphates (OPs) and anti-nerve agents [41]. Thus exposure to chemicals like pyridostigmine bromide (PB) and N, N-diethyl-m-toluamide (DEET) (both of which are neurotoxic) would be likely to produce long-term neurological changes [41] to basal ganglia and brain stem [44]. There is now a growing body of evidence to support this claim. Retrospective epidemiological data has shown a link between self reported levels of exposure to OPs and currently reported levels of symptoms [41]. More compelling biological evidence comes from two recent studies, one using magnetic resonance

(MR) spectrosopy [44] and one examining genetic vulnerabilities (cf. [33,42]). The MR spectroscopy study showed neuronal damage in the basal ganglia and brain stem (5–20% loss). The genetic study showed that ill veterans, and especially those scoring high on Haley et al.'s [38] neurological symptoms complex, were more likely than controls to have low levels of Paraoxonase (PON1) type Q arylesterase [42]. Paraoxonase is a high-density lipoprotein associated enzyme that hydrolyses arylesters and a variety of OPs and nerve agents (e.g., sarin). It follows that an inability to produce sufficient quantities of PON1 Q type would leave veterans particularly vulnerable to the effects of such agents (cf. [33]).

In the case of GWS, neurodegeneration through OP poisoning could have arisen through a variety of routes. First, PB has been found to interfere with controlled (apoptotic) brain cell death, both in vitro and (for up to 30 days) in an in vivo rat model [65]. Furthermore, although the entry of PB into the brain should be minimal, there is controlled experimental evidence that 'stress' (in the rat model induced by restraint and forced swim) can ease the passage of this potential neurotoxin across the blood-brain barrier [45], but see [103]. Also in an animal model, both experimental restraint stress and PB treatment can similarly increase startle responding, consistent with increased anxiety [100].

However, a number of problems have been raised with regard to OP poisoning as a complete account of GWS. First, there was no evidence of acute poisoning in the Gulf (see [29]). Second, a series of studies examining the effects of PB on neuromuscular junctions have shown the effects of PB to be reversible [22] and despite subjective reports of symptoms no pathology has been reported at the neuromuscular level [3]. Third, reported levels of PB exposure have recently been shown to be unrelated to handgrip strength [54]. Finally, a recent study has questioned the synergistic role of stress in relation to OPs passing the blood brain barrier [103].

Notwithstanding these issues the OPIDP model provides some partial account of GWS. However, whilst this theory can account for the persistence of (cognitive) symptoms (related to neurodegeneration), it cannot account for the breadth of symptoms, nor for their temporal variability.

### *4.2. Depleted uranium*

Depleted uranium (DU) is a byproduct of the uranium enrichment process, expressing about 60% of the radioactivity of natural uranium (see [71,84]). It has been argued that exposure to DU particles during the Gulf War is another likely cause of veterans' current illness. A recent report examined the clinical effects of DU in 29 exposed (half with actual DU embedded shrapnel) and 38 non-exposed veterans [73]. A wide range of clinical (e.g., haematological analyses) and neurocognitive assessments (e.g., Wide Range Achievement Test 3, aspects of the Wechsler Adult Intelligence Test - Revised) were performed, with few significant differences shown between the groups. However, there was some evidence that elevated levels of uranium in the urine were associated with a reduction in neurocognitive function, suggesting that perhaps subtle health effects on neurocognition and reproductive health should be explored [73].

As well as these clinical data, the issue of levels of exposure (in terms of numbers affected and dosage) has to be considered. Not all veterans are likely to have encountered DU, in which case DU exposure cannot be a complete account of GWS.

### *4.3. Multiple chemical sensitivity*

This occurs when individuals report a set of allergy-like symptoms, when exposed to everyday volatile environmental agents (e.g., perfumes, glues, paint, cleaning fluids), at levels that most people find tolerable and unlikely to cause illness (see [64]). A number of explanations for MCS have been offered. Among the most extensively researched is an olfactory-limbic neural sensitization model (see [10]). This model suggests that repeated exposures to low levels of environmental toxins induce a process similar to neurological kindling that results in the limbic system becoming sensitized to low dosages of chemicals. In a similar vein, others have described a two-stage process of induction (sensitization) and triggering (generalization) similar to allergic illness [5]. Induction is usually via OPs or carbamates and triggering via generalization to a variety of other chemicals [5].

Bell and coworkers have produced an impressive body of biological and psychological data in support of the sensitization model (see [11–13]). Further support comes from animal models. For example, there are behavioural similarities between Flinder Sensitive Line rats and MCS patients [83] and experimental data showing that rats pre-exposed to formalin vapours demonstrated sensitization to a cocaine challenge [106].

An alternative, although not mutually incompatible, account of MCS is based on Pavlovian associative learning (see [19,102,119]). A series of experimental studies in humans have shown that an odour (e.g., ammonia: conditioned stimulus, CS), previously paired with illness induced by $CO_2$ enriched air (unconditioned stimulus, UCS), will on later presentation elicit the same pattern of somatic symptoms (conditioned response, CR) as seen for $CO_2$ (e.g., dizziness). This effect is known to generalize to other psychologically similar odours (e.g., other unpleasant but not pleasant odours) and to persist over long time durations [19]. However, the conditioning effect was most prominent for respiratory symptoms (e.g., fast breathing, tight chest), supporting the view that a Pavlovian mechanism might support the reporting of allergic type symptoms in particular contexts (e.g., [78]). On balance, it seems likely that the basic biological mechanism for MCS is likely to include sensitization, with Pavlovian conditioning providing a mechanism for generalization.

Does MCS offer a good account of GWS? On the face of it, it does in that it can account for the breadth of symptoms, their persistence and (in principle) their temporal variation. Epidemiological work has demonstrated that deployed veterans are twice as likely to meet the criteria for MCS than are non-deployed veterans (see [14]). However, a number of methodological problems have been identified with the reported literature on MCS and GWS [87]. Specifically, there are concerns about the following: (1) not all studies apply strict criteria for the presence of MCS, with some just assessing a more broadly defined general chemical intolerance (i.e. merely feeling ill when certain chemical smells are present does not necessarily reflect MCS), (2) co-morbidity factors are not assessed, (3) pre-war baseline data are not obtained, (4) not all studies have used reliable and valid assessment methods, and (5) appropriate comparison groups are not always used [87]. Furthermore, the sample sizes in the studies examining MCS in GWS vary from 41 through to 1000s. The highest prevalence rate of 86% was reported for a study with 24 cases and 17 controls and assessed chemical intolerance rather than MCS (see also [75]). By contrast, larger epidemiological studies of Gulf war veterans, where systematic sampling error is likely to be reduced, report prevalence rates of less than 1% for chemical intolerance and 2–6% for MCS (see [87]). On balance the evidence suggests that the prevalence rates among Gulf War veterans are too low to account for the number of veterans suffering from GWS. In fact the prevalence figures would be even lower if corrections for sensitivity and specificity in the measures were applied (cf. [38]).

### 4.4. *Summary of neurological accounts*

There is some evidence for CNS damage, at least in a small proportion of vulnerable veterans, which might be attributable to OP poisoning or neurological insult from DU exposure. In any case, whilst neurological damage on its own can account for the persistence of smaller set of cognitive impairments, it is an insufficient account of the breadth of symptoms seen in GWS, nor can it account for their variability. Similarly, the prevalence rates for MCS are not sufficient to account for the number of veterans reporting GWS.

## 5. Immunological mechanisms

Recent research associated with GWS has explored potential immunological accounts. Is the immune system of those with GWS in some way altered? In terms of basic immunological mechanisms some recent work has focused on the use of squalene as an adjuvant [4]. More psychoneuroimmunological accounts are discussed later.

### 5.1. *The squalene hypothesis*

Squalene is a non-steroidal precursor to cholesterol and produces a multi-system pattern of symptoms similar to that seen in GWS. Asa and coworkers [4] have demonstrated that (in their sample) all (or nearly all) Gulf War veterans who reported being ill (both deployed or not deployed) tested positive for anti-bodies to squalene. By contrast, not a single self-reported healthy Gulf War veteran tested positive for squalene. The argument is that those serving in the Gulf would have been vaccinated with squalene as an adjuvant, but this only really works if squalene administration can be shown to predict symptom levels. A further issue for the squalene account concerns the time interval for symptom onset. These authors reported that one of their positive controls (a National Institute of Health volunteer who received a squalene based adjuvant) became ill after three weeks [4]. However, the majority of veterans reporting GWS describe the onset of symptoms as being years rather than weeks after their return from the Persian Gulf. The issue of variable time delays between squalene administration and symptom onset needs to be addressed by any complete account of GWS. Finally, mild reactions for squalene in blood donors and CFS patients have also been reported [4].

These objections aside, since squalene can produce symptoms ranging from rashes and fatigue to headaches, this account has the potential to cover some of the breadth of symptoms reported in GWS, but not necessarily their persistence or any temporal variability.

## 6. Behavioural mechanisms

It has also been suggested that the experience and reporting of symptoms is mediated by basic Pavlovian mechanisms. Indeed, the work cited earlier in support of a Pavlovian account of MCS (see [19,102]) indicates that the experience of symptoms and their subsequent reporting can be learned and maintained behaviourally, especially in response to odour triggers.

Similarly, rats show ‘bait shyness’ even over long delays between the experience of food and illness, that typically result in complete avoidance of the food subsequently (making them very hard to poison). In human subjects, the extent of this phenomenon has been documented in a questionnaire study (of a sample of 517 undergraduates, 65% reported at least one aversion [66]). Such effects can be quite prolonged so that it requires a motivated effort to overcome initial nausea in order once more to be able to enjoy a particular food or drink. That is everyday experience would suggest that such reactions do not readily spontaneously disappear. However, the robustness of the effect is not necessarily due to a failure to extinguish in the conventional learning theoretic sense because the memory of illness can be sufficient to prevent subsequent exposure to the taste CS in question (and of course exposure is required for extinction to proceed). It follows, therefore, that limbic system structures involved in memory, may be implicated in this type of learning (see below).

In short, an associative mechanism based on flavour aversion could support the maintenance of symptom reporting and explain temporal variability (odour retriggering symptom reporting). However, without further elaboration, an associative account like this cannot account for the breadth of symptoms observed.

## 7. Psychoneuroimmunological mechanisms

Psychoneuroimmunological (PNI) accounts have focused on a role for cytokines in GWS. In particular, they have been based on (1) the relative balance between the Th1 (T-helper-1) and Th2 (T-helper-2) cytokine profiles [93] and (2) bio-associative processes involving IL-1 (see [29]). These models are discussed below, but it is first necessary to discuss the general functions of the cytokines and the Th1/Th2 profiles.

### *7.1. Cytokines and the Th1-Th2 seesaw*

Two types of T helper (Th) response have been recognized in immunology: Th1 and Th2 (see [26,90–92]). These two types of Th response are characterized by different patterns of cytokine activity. The Th1 response is mediated by the cytokines interleukin-2 (IL-2), tumour necrosis factor beta (TNF-$\gamma$) and gamma interferon (INF-$\gamma$). These Th1 cytokines stimulate natural killer (NK) cell and cytotoxic T lymphocyte (CTL) activity. They also activate macrophages and thus promote macrophage mediated inflammatory responses. The cytokine IL-1 is one of the main pro-inflammatory cytokines produced by macrophage activation. The Th1 profile is associated with fever, sickness behaviour, inflammatory responses and autoimmune diseases. The Th2 profile is characterized by the cytokines: IL-4, IL-5, IL-6, IL-10 and IL-13. These stimulate antibody production, in particular immunoglobins A and E (IgA and IgE). The Th2 cytokines also stimulate mast cell growth and eosinophil activation. Overactivity of the Th2 profile is associated with allergic illnesses such as asthma and Th2 activity can also produce inflammation via IgE. The Th1 and Th2 responses counter-regulate each other with INF-$\gamma$ inhibiting Th2 responses and IL-4 and IL-10 inhibiting Th1 responses. Finally, the distinction between Th1 and Th2 responses is very similar to the distinction between cellular immunity (Th1) and humoral immunity (Th2). However, although evidence tends to suggest that the Th1 and Th2 profiles are fairly distinct, there are a group of cells termed Th0 that can stimulate both Th1 and Th2 activity. Reviews of the distinction between Th1, Th2 and Th0-mediated responses can be found elsewhere [26,90–92].

### *7.2. Th2 dominance hypothesis*

Rook and Zumla [93] have put forward a hypothesis that GWS (like CFS) is due to a shift in the Th1/Th2 balance towards a Th2 profile. They argue that 5 conditions present in the Gulf support this: (1) pertussis, a potential Th2 adjuvant, was used, (2) a large antigen load tends to produce Th2 dominance, (3) some of the vaccines used were Th2 inducing (e.g., anthrax, plague), (4) increased cortisol levels due to the stress of battle would favour a Th2 profile and (5) OPs inhibit IL-2 (a Th1 cytokine) driven activity. This account is categorized under PNI because it relies on stress (psychological), OPs (neurologically active) and immunology (Th1/Th2 profiles).

While the argument in favor of a shift towards a Th2 profile is a convincing one, there are still a number of problems with this account. First, a Th2 profile tends to be associated with symptoms of allergic illness. There are reports that veterans show no higher incidence of allergic illness and symptoms than do controls (e.g. [32]). Second, three recent empirical studies have examined a variety of immunological factors (including cytokine profiles) in veterans' groups. One study found no evidence for a Th2 shift [104]. Others have demonstrated that (compared to controls) veterans with CFS showed a pattern of cytokine activity that was consistent with a Th1 rather than a Th2 profile [123]. By contrast, it has been shown that veterans with a diagnosis of PTSD ($N = 3$) were more likely to have reduced cellular (cf. Th1) immunity compared to veterans without a diagnosis of PTSD [27]. However, the Th2 dominance hypothesis relates primarily to CFS and GWS versus controls, so the Everson et al. [27] study does not make the appropriate comparison because they only used subgroups of veterans (with and without PTSD). Chronic stress shifts the immune system towards a Th2 profile (see [26]) and it is therefore not surprising that veterans with PTSD (i.e. under chronic stress) have a Th2 shift. Thus on balance, the current immunological evidence most clearly supports a shift in the cytokine balance towards a Th1 (rather than a Th2) profile in GWS [123]. But whilst immunological accounts such as this may be able to offer an explanation of the symptom diversity, they are less well equipped to explain symptom persistence.

### *7.3. IL-1 and a bio-associative account*

One account that cuts across these different lines of investigation is Ferguson and Cassaday's [29] bio-associative model. This integrates psychological (stress), behavioural (Pavlovian conditioning), neurological (effects of stress and immunological parameters on the CNS) and immunological factors (an IL-1- based sickness response) into a single account of GWS.

The cytokine IL-1 is pro-inflammatory and produces a spectrum of symptoms referred to as the 'sickness response' (fever, fatigue, memory and concentration problems, anorexia, sleep problems, sexual difficulties, depression, HPA activation, see [6,46,58,69,70,117]). Based on the high level of correspondence between the IL-1 induced sickness response and the symptoms seen in GWS, it has been suggested that the sickness response provides a likely basis for the spectrum of symptoms seen in GWS [29]. On this account, the various physiological (e.g., vaccines) and psychological (e.g., stress) challenges present in the Persian Gulf were sufficient to produce a (primary or unconditioned) sickness response [69]. It is further argued that the persistence of symptoms in GWS can be accounted for by Pavlovian bio-associative conditioning of the IL-1 sickness response to smells present in the Gulf (e.g., petroleum, oil fumes) that are also likely to be present in the home environment [29]. Once the sickness response (unconditioned response, UCR) has become associated with smells (CSs, e.g. petrol) that are present at the same time, later exposure to such CSs in the home environment will then produce the associated CR (the sickness response).

While similar in conceptualisation to the Pavlovian account of somatic illness described for MCS [19], this model provides a mechanism whereby environmental triggers could modulate immune responses [29]. However, although the model offers a PNI account of GWS, in that the detection of, and reaction to, environmental triggers will inevitably involve the CNS, such non-specific symptoms are not 'psychologically-induced' in any conventional sense. Through classical conditioning, environmental triggers can have automatic effects on a range of biological systems. These effects are involuntary, typically occur without any conscious awareness and the physiological reactions so produced can be identical to those produced by the original challenge. In brief, the following kinds of evidence support the bio-associative account [29] outlined above: (1) the symptoms of the IL-1 induced sickness response show a high correspondence with the spectrum of symptoms seen in GWS (cf. [46]); (2) in general, there is good evidence that immune system parameters are conditionable [1]; (3) specifically, the neurological circuitry mediating IL-1 responses is conditionable and involved in taste aversion [34] with the bi-directional brain to immune system link mediated by the vagal nerve ([116], see [69] for a review); and (4) under experimental conditions, IL-1 can be used as a UCS to produce flavour aversion [34].

A number of pieces of recent evidence also lend support to this bio-associative account. First, there is now some evidence to support a Th1 shift in the cytokine profile of veterans with GWS (see [123]). IL-1 is produced primarily by macrophages and, although not a defining cytokine, is associated with the Th1 profile. Second, smells and taste, identified by veterans as present in the Persian Gulf, should be associated with current symptom levels. Consistent with this, it has been shown that (once levels of battlefield stress have been controlled for) retrospective recall of environmental exposures is significantly associated with current levels of reported physical symptom reporting [86]. For example, vehicle exhaust was associated with cardiac (e.g., chest pains) and neurological symptoms (e.g., headaches, numbness in limbs, dizziness). Similarly, smoke from tent heaters was associated with cardiac, neurological and pulmonary (e.g., shortness of breath, common cold or flu) symptoms. In addition, it has been shown that self reported war time environmental exposures (e.g., car exhaust, tent heaters etc.) predicted physical functioning in a sample of Gulf War veterans (fatigued versus non-fatigued) [31]. Others have found that current reports of severe GWS are associated with, amongst other things, retrospective reports of having come into contact with smoke or crude oil from oil well fires [80].

Such results mean that likely odour CSs (required by the bio-associative model of GWS [29]) are demonstrably related to symptom levels. It might be argued that these associations are equally supportive of an account of GWS based on MCS. However, as previously described, (see *Multiple chemical sensitivity*, above), the prevalence rate for MCS in veteran samples is not sufficient to account for the numbers reporting symptoms of GWS. Furthermore, the bio-associative model does not require, as an account based on MCS does, that these odours (the CSs) be the cause of GWS, merely that they become associated with the IL-1 sickness response. This matters because some of the odours mentioned (e.g., from car exhaust or tent heaters) are (in the absence of sensitization and/or associative effects) unlikely to cause illness. More importantly the odours identified were likely to have been experienced in the home environment before deployment in the Persian Gulf, so it is reasonable to assume that they were not already associated with feelings of illness. Evidently the physiological effects of such odours for some reason changed during service in the Persian Gulf and the bio-associative account would suggest that this change was due to associations being made with the IL-1 sickness response [29].

Finally, there is evidence that the experience of side effects from the vaccines used predicts current levels of symptom reporting [47]. Such side effects could be part of an IL-1 sickness response and as such would have contributed to the UCS required by the associative mechanism described by Ferguson and Cassaday [29].

### *7.4. Summary of the PNI accounts*

The PNI accounts probably offer the most promising avenues to explain the patterns of symptom reporting seen in GWS. Only further research will tell us whether a Th1 or a Th2 shift offers the best explanation of the underlying immunological processes. Irrespective of the direction of the underlying immune shift, the bio-associative effects could explain the breadth of symptoms in GWS, their persistence and temporal variability. Finally, they offer testable hypotheses, relate current symptoms to experiences in the Gulf and offer a plausible biological mechanism to account for the symptom diversity.

## 8. Summary of explanations of GWS

A variety of theories of GWS have been presented and the following conclusions can be drawn. First, there is limited evidence for neurological damage and this may be associated with OP poisoning. Second, psychiatric co-morbidity is present (particularly depression) but this can only account for a small proportion of the symptoms of GWS. Third, explanations based on PNI appear promising and make clear alternative predictions that are testable. For example, some suggest that there should be a Th2 shift [93] whereas others [29] suggest that there should be a Th1 shift. Finally, stress has a role in many of these models but its role is probably indirect. The implications of these issues are taken up below.

## 9. Cytokines, IL-1, stress, the brain and neurological damage

Based on the above review it is argued that cytokines and in particular IL-1 may have an important role to play in GWS. The biological basis for this would be that IL-1 produces a sickness response. Furthermore, it has been argued that 'stress' may have had a role to play initially as a co-factor in setting conditions that would make the veterans more vulnerable to biological challenges. This final section, therefore, reviews the current literature on the cytokines (in particular IL-1) and how in combination with stress these may influence long term disease and the onset of neurological disorders (e.g., Alzheimer's disease).

The role of psychological stress is included in many of the explanations of GWS described above. However, it is useful when considering stress to differentiate between acute and chronic stress. Whether or not the effects of stress on the immune system are suppressing or enhancing will depend on both the immune parameter being studied and the type of stress (see [24, 26]). Evans and coworkers [26] suggest that acute stress may lead to an enhanced non-specific immune response (e.g., mediated by IL-6, which could lead to increased levels of IL-1) and potentially, increased levels of brain IL-1. Acute stress, in some species at least, may also make the blood-brain barrier more susceptible to these effects (cf. [45,103]). In addition, there is evidence that a single exposure to IL-1 can sensitize HPA axis activity by changes to the hypothalamic control of adrenocorticotropic hormone (ACTH) leading to an increased dominance of vasopressin secretion instead of corticotropin-releasing factor (CRF) [48,97, 98]. It has also been suggested that stress can prime delayed macrophage activity [69]. Thus stress could initiate increased levels of IL-1 (and the sickness response) and in turn IL-1 could sensitize the HPA axis (and again such effects would be exacerbated by a leaky blood-brain barrier).

Were soldiers in the Persian Gulf exposed to acute or chronic stress? In animal work an acute stressor is typically a short duration foot-shock or handling, whereas repeated administrations of foot-shock would be classified as chronic stress. In humans, cognitive appraisals are believed to underlie perceptions of stress [63] and as such the distinction between acute and chronic stress becomes blurred. The soldier in the Persian Gulf would be likely to have anticipated a number of possible negative events (seeing casualties, gas and scud attacks etc.) but may have experienced only one such event. In this case there would have been an acute stressor embedded within an ongoing background of chronic stressors. In humans, there is evidence that the experience of the acute stressor of taking an exam combined with the anticipation of approaching the exam can lead to a Th1 dominant cytokine response [68]. If (against a generally stressful background) the experience of an acute stressor can lead to a Th1 cytokine profile, with IL-1 potentially sensitizing the HPA axis, what further health implications would follow if such a system were self-sustaining and prolonged by Pavlovian associative processes?

There are cytokine receptors in a variety of brain regions and IL-1 in particular has been linked to neurodegenerative disease. For example, the IL-6 receptor is found, in the CA1-CA4 regions and the dentate gyrus of the hippocampus, the hypothalamus and the piriform cortex [99], whilst IL-1 seems to have the hypothalamus as its major site of action (see [95]). There is evidence that the cytokines exert diverse actions in the CNS and (in particular) they have been implicated in responses to disease and injury that have a neuronal component to their biological basis, providing a signalling method to and within the brain [94,95]. Thus, for example, whilst in the healthy brain (in the absence of inflammation), CNS IL-1 and IL-6 show controlled expression in response to peripheral immune challenges, in the diseased brain (after brain damage, e.g., due to OP poisoning), their synthesis is increased and this results in immune reactions, gliosis and neuronal growth [95,99]. As would be expected, cytokines also produce psychological effects. For example, in the rat, IL-1 inhibits long-term potentiation in the hippocampus and intracerebroventricular injections of IL-1 have been found to impair spatial navigation learning measured in the Morris water maze [82]. Similarly IL-1 has been implicated in a number of neurodegenerative diseases such as Alzheimer's disease, Parkinson's disease, multiple sclerosis, and epilepsy (see [89,95,110]). However, the exact causal nature of this relationship is yet to be established. IL-1 levels are also increased during depression [69] and in attempted suicide cases [77].

It follows that if an IL-1 response is involved in GWS, with reactivation and amplification through an associative mechanism, then long-term neurodegenerative diseases should show a higher prevalence in veterans with GWS. However, this conclusion must be seen as speculative as work establishing the causal role of IL-1 in neurodegenerative disease is at an early stage, as is work on the immunology of GWS.

## 10. General conclusions

It is argued that a PNI account of GWS based on a Pavlovian association of an IL-1 induced sickness response to smell may account for the breadth, persistence and variability of GWS and perhaps other functional syndromes. Future research needs to include more small scale theoretically driven studies, focusing on patterns in the report of symptoms, their environmental triggers and immunological correlates, with ERP and MRI scanning studies to examine neurological functioning and how this may change in response to immunological challenges.

Finally, the very brain structures that are damaged by stress (and, as we have seen, IL-1 can contribute to neurodegeneration) are part of the limbic system and this is implicated in the mediation of the bioassociative effects proposed to modulate nonspecific illness. For example, the neural circuitry for taste aversion is known to include both insular cortex and amygdala, and amygdala lesions can also disrupt the acquisition of conditioned immunosuppression [88]. However, we would not expect (for example) hippocampal damage necessarily to impair memory for the bioassociative triggers that activate the limbic system because the relevant associations may be non-declarative [20,23], consistent with the involuntary nature of such effects.
